# Abnormal glycosylation in glioma: related changes in biology, biomarkers and targeted therapy

**DOI:** 10.1186/s40364-023-00491-8

**Published:** 2023-05-26

**Authors:** Juan Yue, Roujie Huang, Zehao Lan, Bo Xiao, Zhaohui Luo

**Affiliations:** 1 Department of Neurology, Xiangya Hospital, Central South University, Changsha, 410008 Hunan China; 2 Xiangya School of Medicine, Central South University, Changsha, 410013 Hunan China; 3 National Clinical Research Center for Geriatric Disorders, Xiangya Hospital, Central South University, 87 Xiangya road of Kaifu district, 410008 Changsha, Hunan China; 4 Department of Obstetrics and Gynecology, Peking Union Medical College, Peking Union Medical College Hospital, Chinese Academy of Medical Science, Shuaifuyuan No. 1, Dongcheng District, 100730 Beijing, China; 5 Clinical Research Center for Epileptic disease of Hunan Province, Central South University, 410008 Changsha, Hunan P.R. China

**Keywords:** Glycosylation, Glioma, Cancer progression, Biomarkers, Targeted therapy

## Abstract

Glioma is a rapidly growing and aggressive primary malignant tumor of the central nervous system that can diffusely invade the brain tissue around, and the prognosis of patients is not significantly improved by traditional treatments. One of the most general posttranslational modifications of proteins is glycosylation, and the abnormal distribution of this modification in gliomas may shed light on how it affects biological behaviors of glioma cells, including proliferation, migration, and invasion, which may be produced by regulating protein function, cell—matrix and cell‒cell interactions, and affecting receptor downstream pathways. In this paper, from the perspective of regulating protein glycosylation changes and abnormal expression of glycosylation-related proteins (such as glycosyltransferases in gliomas), we summarize how glycosylation may play a crucial role in the discovery of novel biomarkers and new targeted treatment options for gliomas. Overall, the mechanistic basis of abnormal glycosylation affecting glioma progression remains to be more widely and deeply explored, which not only helps to inspire researchers to further explore related diagnostic and prognostic markers but also provides ideas for discovering effective treatment strategies and improving glioma patient survival and prognosis.

## Background

Glioma is among the most frequently occurring primary brain tumors and accounts for ∼80% of primary malignancies in the central nervous system (CNS) [1]. Numerous studies have revealed a strong correlation between glioma tumorigenesis and genetic alterations, such as mutations in the isocitrate dehydrogenase (IDH) gene and codeletion of chromosomes 1p and 19q [2, 3]. Notably, the new World Health Organization 2021 classification for glioma brings IDH mutation status and 1p/19q codeletion as criteria to categorize glioma subtypes. In addition, traditional therapies (e.g., surgery, chemotherapy, and radiotherapy) have not established significant improvements in prognosis for glioma patients, and new cancer treatments (e.g., novel molecular-based targeted therapy) are needed. Therefore, distinguishing molecular alterations that occur in glioma may shed new light on the diagnosis, prognosis and precision medicine strategies.

Although studies regarding the impact of DNA mutations on tumor progression provide genetic insights into cancer biology, the crucial role of epigenetic alterations in the development and treatment response of gliomas has recently attracted much attention [4–6]. Protein glycosylation is one of the most prevalent types of posttranslational modification, with over 50% of the human proteome estimated to be glycosylated [7]. In the secretory pathway, glycosylation is categorized into two forms on the basis of the linkages between oligosaccharides: N-linked and O-linked glycosylation. Glycosyltransferases and glycosidases are the main enzymes that orchestrate the successive processes of glycosylation. The glycosylation status of tumor-related factors influences the behavior and biological features of the neoplasm (tumorigenesis, invasion, and metastasis) [8, 9]. Overexpression of MUC4, a highly O-glycosylated protein, was observed in glioblastoma (GBM) cell lines and may take part in processes that promote GBM cell invasion and proliferation through upregulation of epidermal growth factor receptor (EGFR) [9]. N-acetylglucosaminyltransferase-V (GnT-V), whose catalytic products are branched β1,6-N-acetylglucosamine (GlcNAc) structures related to tumor metastasis, is correlated with effects of focal adhesions and tumor invasion [8, 10]. Further investigation of the relationship between the protein glycosylation status and glioma is of great significance for the diagnosis and treatment of glioma.

In the following work, we first describe the current understanding of glycosylation modifications, including N-linked glycosylation and O-linked glycosylation, sialylation, and fucosylation, and their implications for the progression of neoplastic diseases. Apart from summarizing the emerging evidence for glycosylation in glioma, we talk about how aberrant glycosylation mechanism is correlated with the proliferation and migration of glioma. Considering glycosylation alterations and the aberrant expression of glycosylation-associated enzymes (e.g., glycosyltransferase) in glioma, potential biomarkers and therapeutic targets are finally discussed. This review aims to yield a profound understanding of how abnormal glycosylation conduces to the proliferation, invasion and metastasis of glioma and to inspire novel strategies utilizing these changes in the advancement of biomarkers and targeted therapy.

## Introduction to glycosylation

At present, many kinds of glycosylation process have been identified in mammalian species. In this paper, N-linked and O-linked glycosylation are mainly discussed according to the classification of linkage relationships between oligosaccharides. In addition, sialylation and fucosylation can be added to both and are also included. These glycosylation patterns are closely associated with glioma progression.

### N-linked glycosylation

N-linked glycosylation is one of the most general synergistic/posttranslational modification in eukaryotes; that is, oligosaccharides attach to nascent proteins by forming N-glycosyl bonds between monosaccharides and asparagine residues of the consensus sequence Asn-X-Ser/Thr/Cys, which has a profound impact on protein folding, oligomerization, quality control, and physiological function [11, 12].

**Fig. 1:**
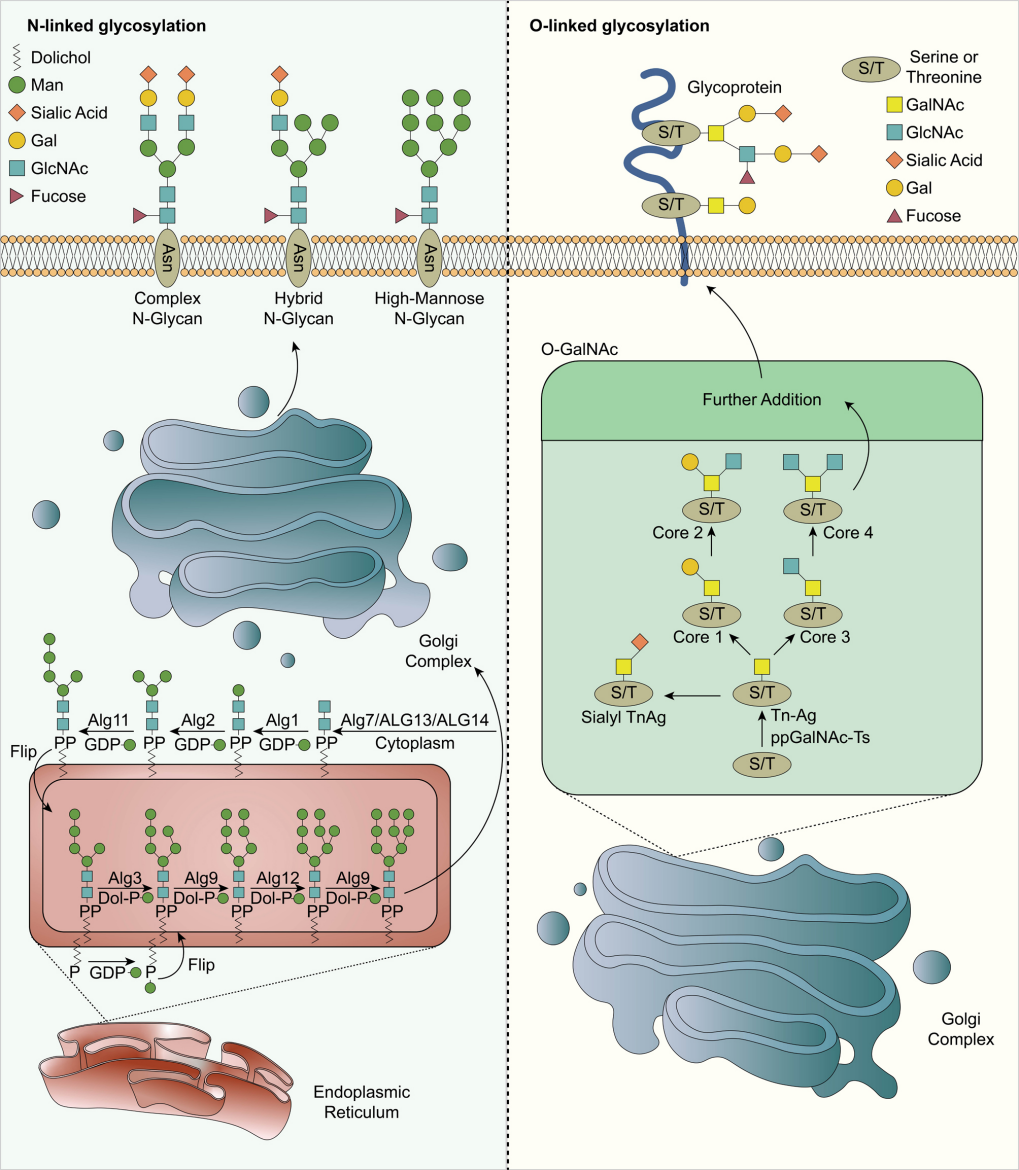
The process of N-linked glycosylation and O-GalNAc glycosylation. The processing of N-glycans begins with an LLO synthesized by linking the cytoplasmic face oligosaccharide intermediate precursor of the ER with dolichol, and the approximate steps are shown in this figure: under the replacement of multienzyme complexes and three mannosyltransferases, one GlcNAc and five Man residues are sequentially transferred from UDP-GlcNAc and GDP-Man to the substrate to generate Man5GlcNAc2-P-Dol.Next, under the action of invertase, LLO undergoes transbilayer translocation across the ER membrane and successively completes four mannosylations in the ER lumen to produce Man9GlcNAc2-P-Dol, and under OST catalysis, the oligosaccharide fraction of LLO is transferred to newly synthesized proteins containing the Asn-X-Ser/Thr (X is any amino acid except Pro) sequence. N-glycosidic bonds are formed between the terminal carbon of GlcNAc and the nitrogen atom of the asparagine side chain of the protein to synthesize N-glycoproteins. Further, N-glycoproteins controlled by ER quality leave the ER, enter the Golgi complex, and are additionally modified to form complex N-glycoproteins that are ultimately transported to the cell membrane. O-GalNAc glycosylation is initiated by GalNAc-Ts catalyzing the addition of GalNAc to peptides, roughly as follows: GalNAc residues are transferred from UDP-GalNAc to the Ser/Thr side chain catalyzed by GalNAc-Ts, and individual GalNAc residues are linked to Ser/Thr α-linkages via αO-glycosidic bonds to form Tn antigens, and subsequently, other glycosyltransferases rapidly extend Tn to other more complex O-glycans. Further, Tn can also be extended to generate four major O-GalNAc cores and four rare cores, core 1, core2, core3, and core 4, as shown respectively, and this type of glycosylation modifies most secreted and cell surface proteins

The processing of N-glycans begins with an lipid-linked oligosaccharide (LLO) synthesized by linking the cytoplasmic face oligosaccharide intermediate precursor of the endoplasmic reticulum (ER) with dolichol, [13, 14] and the specific steps are as follows: (i) on the cytosolic face, the N-acetylglucosamine-phosphate (GlcNAc-P) group of uridine diphosphate-N-acetylglucosamine (UDP-GlcNAc) is transferred to the lipid precursor dolichol phosphate (Dol-P) catalyzed by Alg7/Alg13/Alg14 complex, to generate Dol-P-GlcNAc_2_. (ii) Under the replacement of Alg1, Alg2 and Alg11 mannosyltransferases (MTases), [14–19] mannose (Man) residues are sequentially transferred from guanosine diphosphate mannose (GDP-Man) to the substrate, generating Man_5_GlcNAc_2_-P-Dol. (iii) Under the action of flippase, LLO undergoes translocation across the ER membrane across the bilayer and successively completes four mannosylations in the ER lumen to produce Man9GlcNAc2-P-Dol, [14, 20–22] three glucose residues are added to the end, and the synthesized Glc_3_Man_9_GlcNAc_2_-P-Dol is a specific donor for N-glycosylation [13]. (iv) Under the catalysis of oligosaccharyltransferase (OST), the oligosaccharide moiety of LLO is transferred to newly synthesized proteins containing Asn-X-Ser/Thr (X can stand for any amino acid exclude Pro) sequences. N-glycosyl bonds are formed between the anomeric carbon of GlcNAc and the nitrogen atom of the asparagine side chain of the protein to synthesize N-glycoproteins [13]. The oligosaccharide structure of the N-glycoprotein is modified to Man_8-9_GlcNAc_2_ by α-glucosidase I, α-glucosidase II, and mannosidase [23–27]. After ER quality control, N- glysocylated proteins leave there, enter the Golgi complex, and undergo additional modifications to form complex N-glycoproteins (Fig. 1) [28, 29].

Abnormal N-linked glycosylation is closely associated with cancer [30]. Disturbances in N-linked glycosylation affect the stability of the cadherin-catenin complex and modulate tumor cell‒cell interactions. In human fibrosarcoma, upregulation of GnT-V expression in tumor cells alters N-cadherin function, leading to loss of cell‒cell adhesion and cell invasion [31]. Removal of cadherin-specific N-glycans promotes interactions between cadherin-catenin complexes and stabilizes cell‒cell adhesion [32]. Similarly, N-linked glycosylation of integrins impacts tumor cell-extracellular matrix (ECM) interactions. For example, high N-linked glycosylation affects the adhesion of α_v_β_3_ integrin to its ECM ligand, vitronectin, and promotes melanoma cell invasion [33]. In addition, aberrant N-linked glycosylation leads to deleterious signaling and promotes cancer progression. For instance, in the Wnt/β-catenin signaling pathway, N-linked glycosylation of Wnt ligands, receptors, and E-cadherin promotes catenin expression levels and nuclear translocation, upregulates DPAGT1 transcriptional activity, and promotes tumor metastasis [34]. In summary, aberrant N-linked glycosylation affects multiple processes, including cell‒cell, cell‒ECM adhesion, and signal transduction, ultimately leading to tumor progression and metastasis.

### O-linked glycosylation

O-linked glycosylation is also a unique posttranslational modification that controls important biological processes [35]. It refers to the modification of serine (Ser) and threonine (Thr) residues through O-linked sugar molecules, such as O-N-acetylgalactosamine (O-GalNAc), O-glucose (O-Glc), O-mannose (O-man) and O-fucose (O-Fuc), thereby regulating protein activity or changing protein stability [36, 37]. It is one of the most abundant forms of protein glycosylation that occurs in animals.

For human beings, O-GalNAc is one of the most common O-linked glycosylation modifications and is mainly discussed here [38]. Other types of O-glycans are often found in specific proteins or protein domains, [38] for instance, O-Fuc, which mainly exists in the epidermal growth factor (EGF) domains [39]. O-GalNAc glycosylation refers to glycans via O-GalNAc attached to Ser or Thr residues (GalNAc-α-Ser/Thr) in proteins, usually in clusters, and this type of glycosylation modifies more than 80% of secretory proteins and cell surface proteins, also regarded as mucin-type O-linked glycosylation [36, 40–42]. The process is initiated by the addition of GalNAc to the polypeptide catalyzed by the polypeptide GalNAc-transferase (GalNAc-Ts), [43, 44] which is roughly as follows: GalNAc residues are transferred from uridine diphosphate N-acetylgalactosamine (UDP-GalNAc) to the Ser/Thr side chain, catalyzed by GalNAc-Ts, [45, 46] and a single GalNAc residue is α-linked to Ser/Thr through α O-glycosidic bonds to form Tn antigens, [41] followed by the rapid elongation of Tn by other glycosyltransferases into more complex O-glycans [44]. Tn antigen can be sialylated by α2–6, providing sialyl Tn (Sia α2-6GalNAc α-Ser/Thr); furthermore, Tn can be extended to generate four major O-GalNAc cores and four rare cores, and sialylation can be observed on all types of cores [41]. The most common of them are core 1 and core 2 [47]. The former consists of a galactose (Gal) residue linked to the Tn antigen through β1–3 bonds, which converts Tn into the T antigen, and the latter consists of an GlcNAc residue linked by β1–6 bonds on the basis of the core 1 structure [41]. In addition, GlcNAc-β-Ser/Thr, present in nucleoprotein and cytoskeletal proteins, is different from most other peptide-linked monosaccharides. The β-linked GlcNAc-Ser/Thr is not further extended by other sugars but rather simply leads to monosaccharide modification of the proteins to which it is attached (Fig. 1) [39, 48].

O-GalNAc glycans can crucially involve in various physiological and pathological processes, consisting of tumor growth and progression [41]. Expressed in many tumor cells, O-GalNAc glycans induce various oncogenic signaling molecules in the course of tumor malignant transformation to promote cell‒cell interactions and cell–matrix interactions [49]. Mucin structural changes resulting from O-GalNAc mucin-type glycosylation alter potential ligands for interactions between cancer cells and their microenvironment, which in turn affects cell growth, survival, invasion, and metastasis [50]. Tsuiji et al. used benzyl-α-GalNAc, an O-glycosylation modulator, to inhibit the extension of O-glycans on the cancer-associated glycoprotein dysadherin, thereby inhibiting the stable expression of dysadherin, which in turn leads to upregulation of E-cadherin expression and increased cell‒cell adhesion [51]. In addition, truncated O-GalNAc glycosylation in pancreatic ductal adenocarcinoma has been found to affect two important signaling pathways, AKT/mTOR and RAS/MAPK, which are strongly associated with reduced patient survival and poor prognosis [52]. In O-GalNAc glycosylation modification, because Tn antigen and T antigen are usually masked by the sugar chain structure extended by other glycosyltransferases in normal cells, the expression of Tn antigen and T antigen is believed to be one of the markers of cancer cells [53]. However, the expression of Tn antigen and sialyl-Tn (STn) antigen, is correlated with poor patient survival [54]. Bresalier et al. found that in metastatic colon cancer, mucin sialic acid Tn antigen and sialic acid T antigen increased in metastases, and sialylated epitope antibodies or desialylation produced an effect that inhibited adhesion of metastatic cells to the basement membrane, indicating that increased sialylation of mucin-associated carbohydrates is the most likely feature of metastasizing colon cancer [55]. Gill et al. showed that relocation of GalNAc-Ts leaving the Golgi apparatus for the ER drives high expression status of Tn antigen in malignant tumor cells and 70% of breast cancer, which in turn can stimulate biological behaviors such as cancer cell migration and invasion [44]. In summary, the structural changes of mucin caused by O-GalNAc glycosylation and the high expression of Tn antigen and T antigen can also affect many processes, such as cell‒cell interactions, cell‒ECM adhesion, and signal transduction, ultimately resulting in the malignant progression of cancer cells.

### Sialylation and fucosylation

In addition to the N-linked and O-linked glycosylation processes highlighted above, sialylation and fucosylation can be added to both processes. Among them, sialylation means adding sialic acid to glycan chains grown on glycoproteins or glycolipids, a process that includes 20 kinds of sialyltransferases [56, 57]. Specifically, oligosaccharides and glycoconjugates containing sialic acid are mainly produced by reactions catalyzed by sialylreansferases, which transfer sialic acid from its activated sugar nucleotide, cytidine 5′-monophosphate-sialic acid, to appropriate receptors [58]. N-glycans and O-glycans are usually capped by negatively charged sialic acids [38]. Sialic acid, an acidic monosaccharide, is a group of neuraminic acid derivatives present in cell secretions and extracellular surfaces [59]. Fucosylation, on the other hand, is the enzymatic attachment of L-fucose (also called 6-deoxy-L-galactose) to glycoproteins and oligosaccharides on glycolipids or proteins, a process that is dependent on the activity of fucosyltransferase (FUT) and the expression of guanosine diphosphate-fucose (GDP-Fuc) synthetic enzyme, its donor substrate [60]. Specifically, L-fucose is phosphorylated and conjugated with GDP to produce GDP-Fuc, which is then transported into the Golgi apparatus and ER lumen as a substrate, where proteins on N-glycans can bind to it via FUT in the Golgi apparatus and/or is transferred to protein and form O-fucosylation via two protein O-fucosyltransferase (POFUT) in the ER [61–63].

Research has found that in tumor cells the content of terminal sialic acid and fucose tends to be higher [64]. Sialyl Lewis x (sLe(x)) and sialyl Lewis a (sLe(a)) glycans have been found to be expressed on colon cancer cells with high metastatic properties [65]. Additionally, FUT8, which catalyzes α1,6-fucosylation, is upregulated in malignant tumors, for instance, liver cancer, ovarian cancer, thyroid cancer, and colorectal cancer [66]. Altered sialylation of glycoproteins and glycolipids has been reported to have effects such as promoting tumor proliferation, invasion and metastasis, inhibiting apoptosis, and resisting therapy, as summarized in the relevant literature [57]. For example, in breast ductal carcinoma cells, integrin α_v_β_3_ is sialylated, which promotes their migration and invasion, while absence of sialylation inhibits their metastatic potential [67]. Fucosylation is known to affect cell‒cell adhesion, signaling, and immunosuppression in malignant tumors [68–70]. Lai et al. reported that FUT2 knockdown in T47D, a kind of breast cancer cell lines significantly reduced cell proliferation, adhesion, and tumor formation [68]. Alternatively, Notch signaling is strongly implicated in cancer, whereas O-fucosylation has been found to act as a Notch signaling modulator [69]. According to Huang et al., FUT8-mediated core fucosylation stabilizes the type I transmembrane protein of the B7 immunoglobulin superfamily, B7H3, which of importance for the immunosuppressive function of B7H3 in triple negative breast cancer cells [70].

### Evidence of abnormal glycosylation in gliomas

Different classes of glycosylation processes are discussed above; meanwhile, researchers have demonstrated the presence of altered glycosylation in gliomas from different perspectives and using different methods. Toghi Eshghi et al. adopted matrix-assisted laser desorption/ionization mass spectrometry (MALDI-MS) to image N-glycans in formalin-fixed paraffin-embedded tissue sections and found large differences in glycosylation between tumor tissue and adjacent normal tissue in the mouse brain tumor model: low-abundance N-glycans in tumor cells had higher levels of fucosylation, while high-abundance N-glycans in tumor cells were mainly composed of oligomannose and nonfucosylated complex glycans [71]. However, there was a lack of sialylated glycans in the spectra of this study, and the possible explanation given by the authors is the loss of sialic acid residues during MALDI-MS. Furthermore, Malaker et al. performed peptide-N-glycosidase F (PNGaseF) digestion and matrix-assisted laser desorption/ionization mass spectrometry imaging (MALDI-MSI) on canine glioma biopsies; to identify potential sialylated glycoproteins, they performed microdigestion and manual glycoproteomic analysis of various regions in adjacent tissue sections, demonstrating that sialylated glycans are elevated in canine gliomas and illustrating the complementary role of spatially resolved glycoproteomics in understanding glycosylation dysregulation using MALDI-MSI [72]. In addition, mass spectrometry and high-performance thin-layer chromatography were used to characterize the natural ganglioside mixture from GBM multiforme, corresponding peritumoural tissues and healthy human brain in detail. Ganglioside expression was found to be significantly changed in GBM compared with healthy brain tissue [73]. Gangliosides are salivary glycosphingolipids that are highly abundant in neural tissue, and abnormal glycosylation of glycoconjugates on the cell surface is often considered characteristic of tumor cells [74].

The extracellular matrix often involves in glioma growth, invasion and adhesion [75]. However, few studies have focused on the glycosylation of extracellular matrix components and their biological characteristics in glioma. Sethi et al. performed in-depth glycoproteomic analysis of the matrix and its components, including proteoglycans (PGs) and glycosaminoglycans (GAGs), in control and GBM samples and found that glycosylation was higher and glycosyltransferase and glycosidase expression levels were increased in GBM compared with control samples [75].

The different spatial distributions of glycosylation in normal or pathological brain structures may clarify its role in mediating brain function. The above studies have confirmed the existence of abnormal glycosylation in gliomas using different methods, and its function and biological changes will be reviewed in more detail later.

## Function of glycosylation in gliomas

### Regulating protein function

On the one hand, some glycosylation-related enzymes, such as UAP1L1, have been shown to be upregulated in gliomas; on the other hand, some highly glycosylated proteins have been found. They include P2Y_14_ receptor protein, SIRPα1, and MUC4, which are overexpressed in glioma cells and eventually produce biological effects such as glioma cell proliferation, invasion, and adhesion [9, 76, 77].

Specifically, from the perspective of glycosylation-related enzymes, Peneff et al. found that uridine diphosphate-N-acetylglucosamine pyrophosphorylase-1 (UAP1), which can catalyze the synthesis of UDP-GlcNAc, and in glioma, the expression of its paralog UAP1L1 was significantly upregulated and closely related to poor prognosis [78]. Yang et al. found that the proliferation and colony-forming ability of glioma cells were inhibited by knockdown of UAP1L1,which induced their apoptosis and inhibited their growth [79]. Thus, they proposed that it may lead to glioma proliferation by altering glycosylation condition of some principal proteins.

**Fig. 2:**
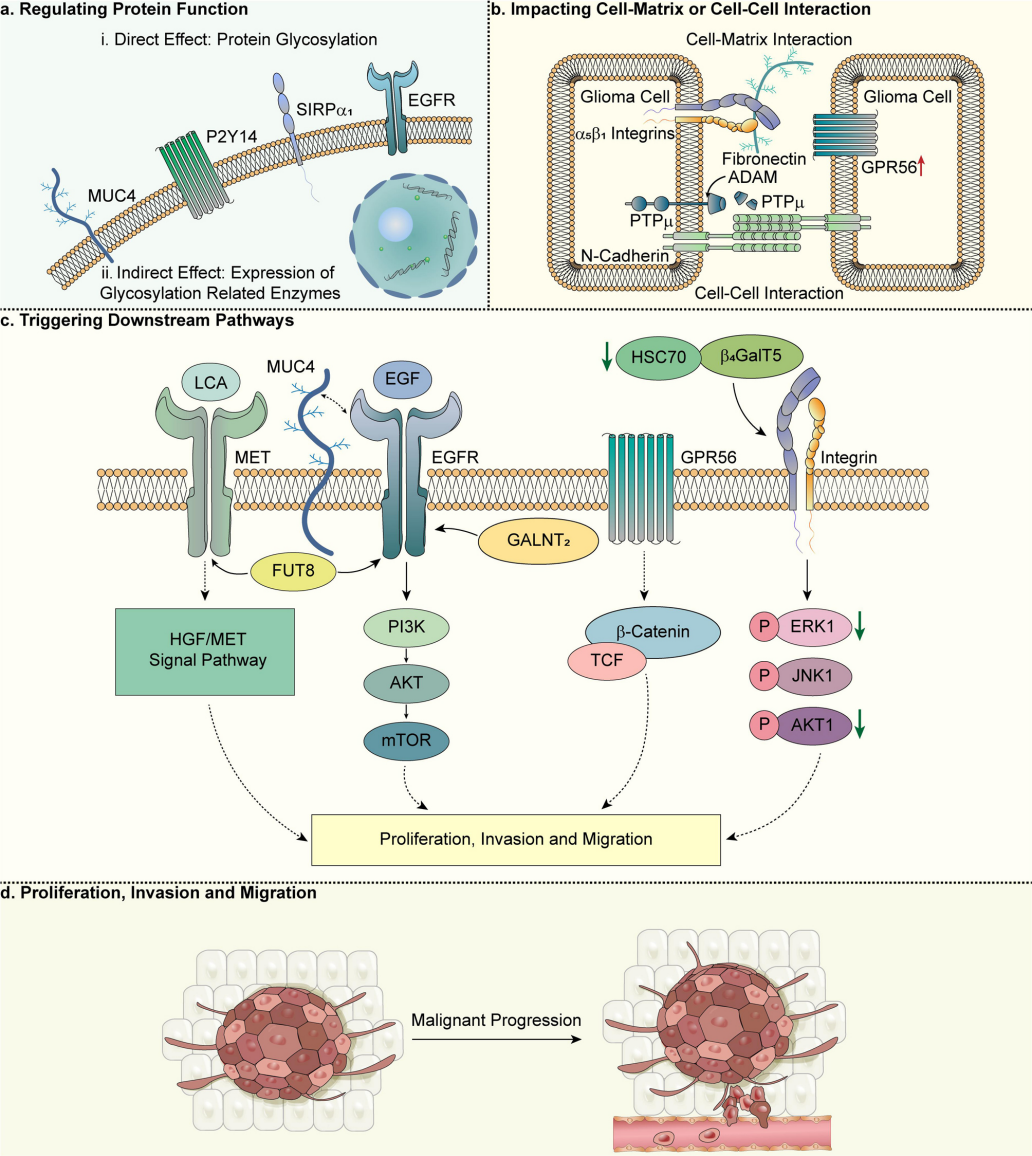
Function of glycosylation in gliomas. Glycosylation impacts on glioma progression include regulating protein function (a), affecting cell‒cell or cell‒matrix interactions (b), and triggering downstream pathways (c), which ultimately produce malignant progression of gliomas (d), and various types of research have gradually advanced. (b) For changes in cell‒cell or cell‒matrix interactions, studies of glioma cell‒ECM interactions have found that gliomas can interact with the ECM through cell surface proteins, including chondroitin sulfate proteoglycans (CSPG) of the lectican family, GPR56, β1 integrins, etc. In addition, N402 glycosylation deficiency disrupts N-cadherin stabilization, ultimately inhibiting cadherin-mediated cell‒cell adhesion and promoting cell migration. PTPµ can be differentially glycosylated, full-length PTPµ produces a larger shed extracellular fragment PTPµ after direct cleavage by ADAM protease, a process that impacts cell‒cell interactions. (c) More specifically, the effect of glycosylation on the malignant progression of glioma can be achieved by triggering downstream pathways. For instance, FUT8 can be involved in altering the fucosylation status of MET and EGFR, and its knockdown or overexpression corresponds to the decrease and increase in the binding of LCA to MET and EGFR in glioma cells, and further, the HGF/MET signaling pathway is significantly activated by it. Whereas down-regulation of GALNT2 expression inhibited O-glycosylation, phosphorylation, and its downstream PI3K/Akt/mTOR pathway of EGFR, overexpression of GALNT2 had the opposite effect. In addition, ectopic expression of the highly glycosylated protein MUC4 regulates EGFR expression. Among other pathways, the ability of GPR56 to activate the β-catenin/TCF pathway may be involved in the transition from benign tumors to invasive metastatic cancers. Downregulation of Hsc70 may influencethe glycosylation and maturation of substrate integrin β1 by regulating the protein folding of β4GalT5, reduce the expression levels of β4GalT5 downstream signaling proteins p-ERK1, p-JNK1 and p-AKT. (d) The effects above can ultimately promote the proliferation, invasion and migration of glioma cells

From the perspective of abnormally glycosylated proteins, according to earlier studies, the application of a panel of N-glycosylation inhibitors and traffic indicated that C6 cell proliferation and adhesion could depend on the expression of glycoproteins containing oligomannans and hybrid N-glycans on the cell surface; meanwhile, the lack of N-glycans or the existence of glucosyl-oligomannosides and the lack of cell surface glycoproteins reduced C6 cell proliferation and adhesion [80]. This study indirectly established a link between N-glycosylation and glioma cell proliferation and adhesion properties using glycosylation inhibitors. Another study showed that in C6 glioma cells, N-glycosylated Gαi/o protein-coupled P2Y_14_ receptor may change its functional activity, in which glycosylated P2Y [14] receptor gets primarily involved with intracellular calcium mobilization, while nonglycosylated P2Y14 receptor is involved in adenylyl cyclase inhibition [76]. In addition, Chen et al. found that underglycosylated SIRPα is expressed in malignant astrocytes, unlike normal astrocytes, and further speculated that it may alter the affinity of SIRPα1 for CD47 (integrin-related protein), its isoforms, or other unknown ligands [77]. Although previous researches have revealed that transfecting SIRPα into U87MG, a kind of GBM cell lines, brings about tumor spreading and migration defects, [81] further observation and exploration are needed to determine the correlation between the malignant progression of astrocytoma cell lines and SIRPα expression (Fig. 2a).

In conclusion, the changes in some protein functions associated with abnormal glycosylation may be closely related to the relevant biological behaviors of glioma cells. The above studies all involve changes in protein function produced by abnormal glycosylation; however, more specifically, the effects of glycosylation-related enzymes or related proteins on cell‒cell interactions, cell‒matrix interactions or downstream cascade pathways remain to be more deeply studied to further confirm the specific mechanism of action behind them or biological behavior changes associated with them, which may reveal more potential value.

### Impacting cell‒matrix or cell‒cell interactions

Gliomas invade normal neural tissue in a unique infiltrative and scattered manner and can overcome cell movement barriers in the CNS [82]. ECM is one of the crucial obstacles to cell motility in all tissues [83]. In the CNS, the ECM predominantly comprises hyaluronan scaffolds with linked glycoproteins and PGs. Typical ECM proteins, for example, laminin, collagen IV, and fibronectin, are restricted to the vascular basement membrane and glial cell boundaries in the adult CNS and are not actually present in the parenchyma [84]. Studies on the interaction between glioma cells and ECM have found that gliomas can interact with ECM through cell surface proteins (including chondroitin sulfate proteoglycans (CSPGs), G protein-coupled receptor 56 (GPR56), β1 integrins, etc.) [82, 85, 86]. The effect of abnormal glycosylation on the invasion and migration of glioma is closely related to the abnormal expression level of glycosylation-related enzymes. Currently, many studies involve aberrant glycosylation affecting glioma cell interactions with the ECM.

The findings of Silver et al. support that the loss of glycosylated CSPG provides favorable conditions for diffuse infiltration typical of high-grade gliomas, i.e., the presence of highly glycosylated microenvironment CSPG is inversely correlated with aggressive features of human gliomas [87]. Brevican is one of five core proteins of CSPG in human glioma cell lines. Several isoforms of brain-enriched hyaluronan binding (BEHAB)/brevican have been reported to interact differently with ECM components or cell membrane components [87]. The isomer B/b_Δg_ generated by differential glycosylation is upregulated in rat and human glioma ECM and may crucially involve in glioma progression. B/b_Δg_ can localize to the extracellular surface of glioma cells, and its overexpression may promote tumor progression by interfering with the normal interaction of BEHAB/brevican to achieve novel cell‒cell interactions conducive to invasion [87]. For glioma cells, the equilibrium between adhesion and separation determines their aggressive biological nature, and tumor migration is influenced by the traction, rejection, and stimulatory properties of cell adhesion receptors and related ligands [85]. GPR56, which colocalizes with α-actinin, is expressed at the leading edge of membrane filopodia and may be involved in cell‒cell or cell‒matrix interactions. Shashidhar et al. demonstrated upregulation of GPR56 in GBM multiforme (Fig. 2c) [85]. In most glioblastoma/astrocytoma samples expressing the GPR56 protein, the amino-terminal domain contains a large number of possible N- and O-linked glycosylation sites, which are similar to mucin-like proteins [85]. In skeletal muscle cells and other nonmuscle tissues, dystrophin-glycoprotein complexes (DGC) connect ECM, such as laminin and PGs, to the actin cytoskeleton [88]. Dystroglycan (DG), a key component of the DGC, is an adhesion molecule comprising α and β subunits. Produced by posttranslational cleavage of a single precursor molecule, it plays an important role in forming stable contacts with ECM molecules. The results of Calogero et al. showed that in some glioma cells, the hyperglycosylated α-DG subunit was significantly reduced [89]. In addition, there is a hypersialylated β1 integrin on the membrane of the human astrocytoma cell line A172 that can heterodimerize with α5 and adhere to fibronectin. However, changes in N-glycans in α_5_β_1_ integrin contribute to altered adhesion properties of tumor cells and tumor formation [86].

In addition, studies have been conducted to investigate the effects of glycosylation-related enzymes on ECM. A key enzyme, sialidase, which controls cellular sialic acid content by removing sialic acid residues from glycoproteins and glycolipids, regulates calpain activity and focal adhesion disassembly as well as the invasive potential of GBM cells and affects cell invasive ability [90]. Evidence demonstrates that Sialidase neuraminidase 3 (NEU3) modulates invasion and migration by regulating calpain-dependent adhesion proteins as well as stabilizes cell adhesion to collagen IV and fibronectin and inhibits cell spreading [90]. In contrast, the structure of polylactosamine β1–6 branched chain N-glycans is catalyzed by β1,3-N-acetylglucosaminyltransferase 8 (β3GnT8), whose expression level is associated with glioma progression and significantly affects cell migration and metastasis [91]. This effect may occur by inducing matrix metalloproteinase-2 (MMP-2) expression. Among them, MMP-2 promotes cancer cell invasion and metastatic spread by degrading type IV collagen, a major component of the basement membrane.

For cell‒cell interactions, cell‒cell communication includes tight junctions and adherens junctions. The former mainly takes responsibility for cell-to-cell communication through barriers, while the latter acts as adhesion modulators between adjacent cells [49]. E-cadherin, a kind of calcium-dependent transmembrane protein, actively modulates cell characteristics of growth, differentiation, motility, and adhesion by the formation of a cadherin-catenin complex between adjacent cells [49]. In gliomas, glycosylation deficiency in one of the three N-glycosylated asparagine residues of N-cadherin, N402, disrupts N-cadherin stabilization and results in its proteasomal degradation, whereas N-cadherin destabilization eventually prevents cell‒cell adhesion and promotes cell migration [92]. Alternatively, a disintegrin and metalloproteinase (ADAM), whose family member ADAM12 is an N-glycosylated protein, is highly expressed in human GBM and may involve in cell‒cell adhesion [93]. Phillips-Mason et al. reported that the cell‒cell adhesion molecule receptor protein tyrosine phosphatase type µ (PTPµ) is differentially glycosylated in GBM cells, and ADAM proteases can directly cleave full-length PTPµ and produce a larger shed extracellular fragment PTPµ [94]. This study supports the “protease storm” theory that aggregation of multiple proteases occurring in cancer cells will reduce the presence of cell‒cell adhesion molecules on the plasma membrane and produce effects that promote cancer cell migration and invasion [94].

In general, this section more deeply discusses the structural and functional changes of related proteins caused by abnormal glycosylation in gliomas, affecting glioma cell‒matrix or cell‒cell interactions, thereby changing glioma adhesion, invasion and migration characteristics, which specifically include changes in related proteins in the ECM or changes in cell surface receptors resulting in their interaction with the ECM. The overview of glycosylation impacting cell‒matrix or cell‒cell interactions is shown in Fig. 2b.

### Triggering downstream pathways

The signaling cascades often discussed in tumor-related studies include Wnt/β-catenin, PI3K/Akt, TGFβ/Smad and Notch signaling pathways, [49]. and changes in glycosylation status on cell surface molecules, transmembrane proteins and growth factors can also affect the proliferation, invasion and other biological behaviors of tumor cells [95]. Aberrant and modified glycosylation triggers downstream pathways, which in turn promote cancer progression, and it is critical to study the specific molecular mechanisms behind it. These are concretely sorted out in Table 1.

**Table 1:** Related pathways and specific effects

Pathway	Enzyme or protein	Category	Specific effects	Reference
RTK related pathway	GnT-III	N-acetylglucosaminyltransfease	Influencing EGF binding and receptor autophosphorylation	[106]
	GnT-V		Influencing PLCγ-PKC pathway	[111]
	GALNT2	N-acetylgalactosaminyltransferase	Influencing PI3K/Akt/mTOR	[114]
	FUT8	Fucosyltransferase	Influencing LCA binding to MET and EGFR	[96]
	MUC4	Mucin	Regulating EGFR expression	[9]
	GOLPH3	Golgi phosphoprotein	Decreasing EGFR proliferation signaling activity	[116]
TGF-β pathway	ST3GAL1	Sialytransferase	Indirectly controlling FoxM1 protein degradation by the APC/C-Cdh1 complex	[117]
MAPK pathway	Hsc70	Heat shock protein	Decreasing the levels of β4GalT5 downstream signaling proteins P-ERK1, P-JNK1 and P-Akt.	[119]
	Advanced Glycation End Products	Glycotoxins	Activating tyrosine kinase and RAS related pathways, inducing the activation of p38 MAPK	[168]

It is currently believed that activation of the receptor tyrosine kinase (RTK)-related signaling pathway is the most general changes in human gliomas, and some researches have shown that RTK activity can be modulated by fucosylation [96]. In recent years, the exploration of abnormal glycosylation affecting downstream pathways in glioma has focused on epidermal EGFR of the RTK family and its downstream pathways, which can also be divided into two parts: glycosylation-related enzymes and glycosylation-related proteins according to different research subjects.

EGFR, also known as ERBB1 or HER1, belongs to the ErbB family with tyrosine kinase function [97, 98]. In addition to EGFR, the family includes three receptors, HER-2/ErBB2, HER-3/ErBB3, and HER-4/ErBB4 [99, 100]. The traditional signaling pathway of EGFR involves the transduction of mitogenic signals through the activation of signaling molecule cascades: EGFR can first bind to ligands such as EGF, transforming growth factor-α (TGF-α), bidirectional regulatory protein, β-cell protein or epiregulin and then it is activated by forming homodimerization or heterodimerization with other ErbB receptors and subsequent tyrosine autophosphorylation [100–102]. Activated EGFR recruits and activates many important signaling molecules, of which the main downstream pathways involve the PLC-γ-CaMK/PKC, Ras/RAF/MEK/ERK, PI3K/AKT and STAT pathways [100, 101, 103]. At present, the above signaling molecule cascades activated by EGFR are believed to be closely related to the activation of genes related to cell proliferation, survival and differentiation [104, 105].

Many studies have shown that glycosylation-related enzymes in gliomas can influence EGFR function. As early as in a previous study, Rebbaa et al. compared the effects of the bisecting structure on cell surface and EGFR expression, EGF binding, receptor autophosphorylation, and cell proliferation in U373 MG cells transfected with N-acetylglucosaminyltransferase III (GnT-III) and controls, respectively [106]. However, because the addition of bisecting GlcNAc branch to N-glycans can be catalyzed by GnT-III, which is generally identified as a metastasis suppressor, [107]. GnT-III overexpression is believed to enhance E-cadherin-related cell‒cell adhesion and downregulate integrin-mediated cell migration, which may help to inhibit cancer cell metastasis; [108] however, GnT-III expression is increased in gliomas, which seems to contradict its effect as a metastasis suppressor, [109] and Lu et al. attributed the controversy to the divergent expression patterns of cellular sialylation [107]. Their studies demonstrate that increased α2,6-sialylation on glioma cell surface may influence the anti-migratory effect of GnT-III, and overexpression of GnT-III significantly inhibits α2,3-sialylation but not α2,6-sialylation [107]. In addition, high expression of ST6 beta-galactoside α2,6-galactoside sialylatransferase 1 (ST6GAL1) may weaken GnT-III action and confer strong metastatic potential to cells, and the counteraction of ST6GAL1 on GnT-III in cell motility may be mediated by many molecules associated with cell adhesion and migration [110]. A detailed description of how α2,6-sialylated N-glycans and bisecting N-glycans influence the signaling pathways regulated by these molecules is needed to better understand the interaction between ST6GAL1 and GnT-III in the regulation of tumor migration. In addition, for another member of the GnT family, GnT-V, which catalyzes the transfer of GlcNAc from UDP-GlcNAc to α1,6-Man residues form a β1,6-branch, the role in tumor metastasis has been reported by many investigators [111]. They believe that it can promote cancer cell metastasis by catalyzing N-linked glycosylation of growth factors and cell surface receptors to regulate EGFR, TGF-β family oncogenes and related signaling pathways [112]. As cell adhesion molecules and tyrosine phosphatases, receptor protein tyrosine phosphatase types (RPTPs) have the ability to affect cell adhesion and cell signal transduction, and their β1,6-GlcNAc-branched N-glycans play an important role in glioma invasion [111]. In glioma cells, PTPµ fragments were found to be increased, whereas PTPµ had 12 potential N-linked glycosylation sites in the extracellular region, which revealed the structural basis of aberrant glycosylation [113]. Aberrant glycosylation has been reported to reduce the phosphorylation activity of PTPµ, promoting glioma cell migration through the PLCγ-PKC pathway [111].

Down-regulation of N-acetylgalactosaminyltransferase 2 (GALNT2) expression can inhibit the proliferation, migration and invasion of glioma cells by inhibiting O-glycosylation, phosphorylation of EGFR and its downstream PI3K/Akt/mTOR pathway, and the opposite effect is observed when GALNT2 is overexpressed (Fig. 2c) [114]. Another study showed that FUT8, an enzyme that catalyzes the transmission of fucose residues from the donor substrate 5’-diphosphate-beta-L-fucose to the reducing terminal GlcNAc of the asparagine-linked oligosaccharide core structure, [115] is an important regulator of malignant features of human gliomas, and its knockdown or overexpression corresponds to a decrease and increase in the binding of lens culinaris agglutinin (LCA) to mesenchymal-epithelial transition factor (MET) and EGFR in glioma cells and affects the migration and invasion of glioma cells [96]. Wei et al. suggested that it could be involved in altering the fucosylation status of MET and EGFR, and the HGF/MET signaling pathway was significantly activated by it. In addition, transactivation of EGFR is also influenced by its expression (Fig. 2c) [96].

In addition to enzymes, some studies have considered the impact of abnormal glycosylated proteins themselves on downstream signaling pathways; for example, as highly glycosylated proteins, MUC4 is overexpressed in GBM cell lines and tissues, while ectopic MUC4 expression can promote GBM cell proliferation and invasion characteristics by regulating EGFR expression (Fig. 2c) [9]. This work may help target novel pathways of MUC4 to undermine signaling cascades involved in GBM proliferation, motility, and invasion. In addition, reduced expression levels of fucosylation and/or sialylation of EGFR following Golgi phosphoprotein 3 (GOLPH3) knockdown decreased EGFR proliferation signaling activity [116]. Datas from Arriagada et al. indicate that in T98G cells, expression levels of GOLPH3 modulate glycosylation of EGFR, affecting its endocytic endocytosis and activation; however, further studies are needed to fully understand the tumorigenicity of GOLPH3 (Fig. 2c) [116].

Among other pathways, as mentioned earlier, the study by Shashidhar et al. illustrates the link between upregulated GPR56 expression in glioma samples and cell‒cell or cell‒matrix interactions, explaining signaling pathways in which there may be an association [85]. Their reporter assays also found that transient overexpression of GPR56 resulted in activation of specific signaling cascades, [85] and activation of the β-catenin/TCF pathway by GPR56 may be correlated with the transition from benign tumors to malignant cancers (Fig. 2c) [85]. In addition to the ST6GAL1 mentioned above, the sialyltransferase gene ST3GAL1 has also been found to be associated with breast, colorectal, and bladder cancers. Chong et al., on the other hand, showed for the first time how ST3GAL1 sialyltransferase is triggered by the TGF-β signaling pathway in a cohort of mesenchymal patients and regulates glioma formation by targeting degradation of the Forkhead box M1 (FoxM1) protein through anaphase-promoting complex/cyclosome (APC/C-Cdh1) [117]. Zhang et al. showed that the effect of CD109 on the TGF-β1 signaling pathway in GBM cells may be related to the glycosylation of the CD109 N-terminal fragment and is cell-type dependent [118]. In addition, β-1,4-galactosyltransferase 5 (β4GalT5) is a member of the β1,4-galactosyltransferase family and is effective in galactosylating the GlcNAcβ1,6Man arm of highly branched N-glycans with glioma characteristics. Sun et al. speculated that Hsc70 may modulate glycosylation and maturation of the substrate integrin β1 by regulating the protein folding of β4GalT5, thus promoting the proliferation of glioma cells [119]. This process may be related to the fact that downregulation of Hsc70 decreased the expression levels of the β4GalT5 downstream signaling proteins p-ERK1, p-JNK1, and p-AKT (Fig. 2c) [119].

According to the discussion above, the processes influencing glycosylation in promoting tumor progression include regulating protein function, affecting cell‒cell or cell‒matrix interactions, and triggering downstream pathways. In conclusion, abnormal protein glycosylation is profoundly rooted in the malignant transformation of glioma cells; in addition to changes in protein function, cell‒matrix and cell‒cell interactions, and invasive tumor behavior resulting from the cascade pathway behind it (Fig. 2d), cancer-related glycan changes has been suggested to also promote cancer invasiveness by triggering anti-inflammatory signaling pathways in tumor-infiltrating immune cells. For example, GBM cells evade the immune system by recruiting tumor-associated macrophages (TAMs), which contain at least one-third of the cells in the GBM microenvironment and promote tumor malignant transformation by creating supportive environment for tumor cell proliferation and migration [120]. In the tumor microenvironment, M1-like TAMs may be involved in inhibiting tumor progression, while M2-like TAMs can promote tumor growth, invasion, angiogenesis and chemotherapy resistance. The hexosamine biosynthetic pathway and O-linked N-acetylosamine transferase-mediated protein O-GlcNAc glycosylation modification activity is increased in M2-like TAMs [121]. These findings may also suggest more possibilities for understanding and interpreting the abnormal glycosylation and malignant behavior of gliomas.

## Biomarker and treatment

### Biomarker

**Fig. 3:**
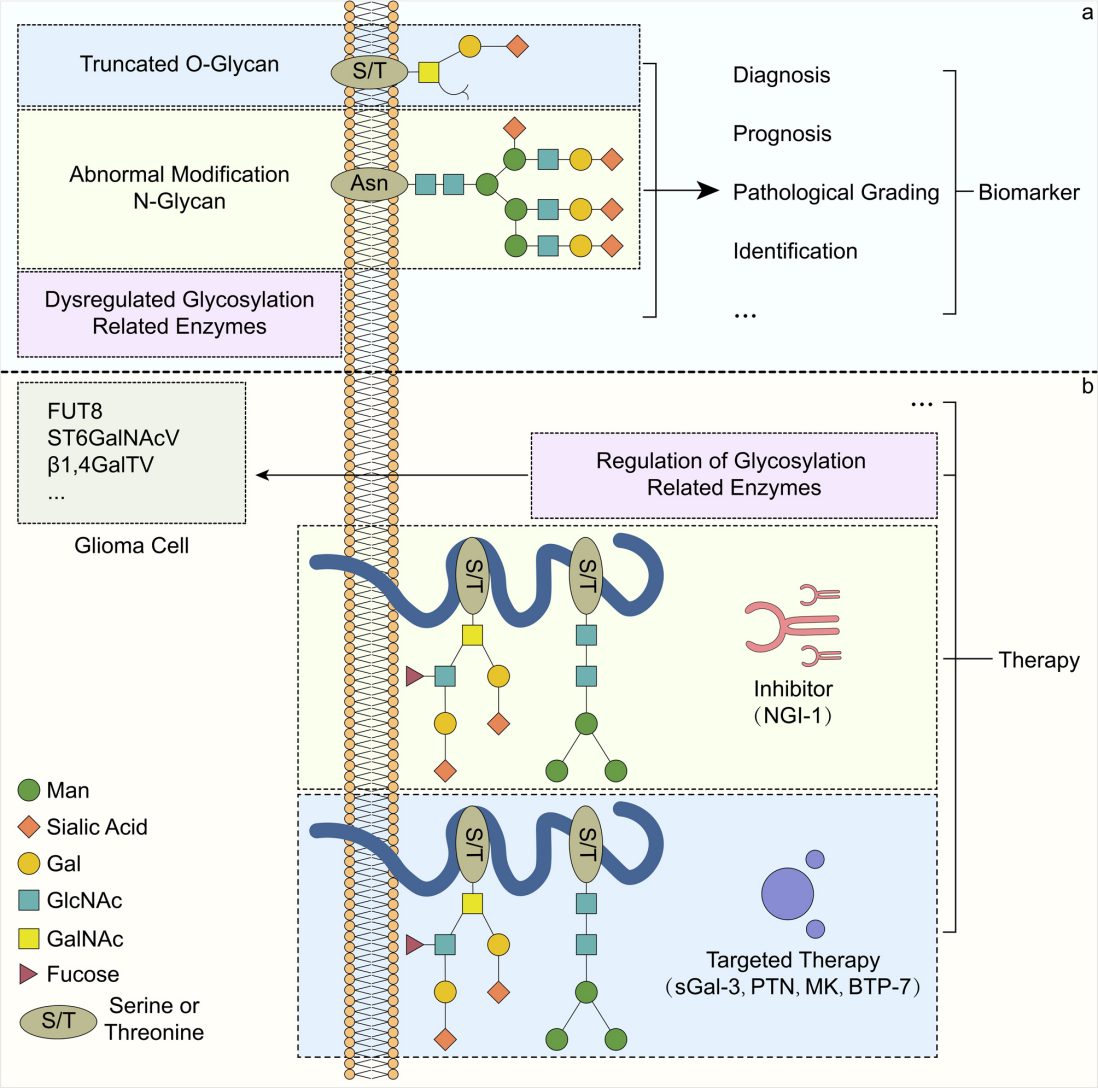
Biomarker and treatment. (a) The changes of glycosylation-related enzymes and cell surface glycosylation in glioma provide an important source of markers for its diagnosis, prognosis, pathological classification, and pathological diagnosis. (b) In terms of treatment, it can be achieved by regulating the expression of glycosylation-related enzymes (such as FUT8, ST6GalNAcV, β1, 4GalT V …), using glycosylation inhibitors (such as NGI-1), and targeted therapy (such as sGal-3, PTN, MK, BTP-7).

As mentioned earlier, glioma-related glycosylation changes are closely related to changes in their biological behavior, and the occurrence of protein glycosylation changes and abnormal expression of glycosylation-related enzymes (such as glycosyltransferasesin gliomas may allow some proteins to be used as novel biomarkers in clinical practice (Fig. 3a). In related efforts, the National Cancer Institute also undertook a program to identify and validate cancer biomarkers associated with glycosylation. Because treatment strategies differ significantly from infection or inflammatory diseases, preoperative diagnosis of glioma is essential, and selection of appropriate markers for testing may even avoid the use of biopsy to diagnose glioma [122]. This section will focus on the possibility and clinical significance of dysregulated glycosylation-related enzymes, glioma-related membrane binding or secreted proteins, and abnormal glycosylation and glycosylation-related genes present in ECM as biomarkers (Table 2).

**Table 2:** Biomarkers associated with abnormal glycosylation

Biomarker	Category	AUCs	Sample	Clinical value	Reference
GnT-III	N-Acetyl-glucosaminyl transferase III	-	Glial tumor cell lines and glial tumors	Diagnosis	[107]
GALNT	N-acetylgalactosamine transferase	AUC_1year_ = 0.881; AUC_3year_ = 0.873; AUC_5year_ = 0.779	LGG	Prognosis	[125]
PSCA	TAA	-	Glial tumor cell lines and glial tumors	Diagnosis	[126]
sPTPRZ	Protein tyrosine phosphatase receptor type zeta	0.9676	CSF	Diagnosis	[122]
PLAUR	Integral membrane protein	CGGA-RNA-seq: AUC_1year_ = 0.7810, AUC_3year_ = 0.8437, AUC_5year_ = 0.8688 TCGA-RNA-seq: AUC_1year_ = 0.8528, AUC_3year_ = 0.8485, AUC_5year_ = 0.8074	Undecided	Prognosis	[127]
gp273	Nucleolin	-	Glial tumor cell lines and glial tumors	A histopathological marker for glioma grading	[128]
dg-Bcan	BEHAB/ brevican	-	Glioma tissue	Distinguishing primary brain tumors of similar histology but different pathologic course	[82]
Galectin-3	β-galactoside-specific animal lectins	-	Glial tumor cell lines and glial tumors	Diagnosis	[169]
TXNDC12	Thioredoxin domains	TCGA-RNA-seq: AUC_3year_ = 0.787, AUC_5year_ = 0.755; CGGA-RNA-seq: AUC_3year_ = 0.748, AUC_5year_ = 0.774	Glioma tissue	Prognosis, glioma pathological grade	[170]
CD133	Pentaspan transmembrane glycoproteins	-	Glioma tissue	Identifying brain tumor stem cells in gliomas	[134, 137]

AUC: the area under the ROC curve; TAA: tumor-associated antigen

Many dysregulated glycosyltransferases and glycosidases produce glycan structures that are robust biomarkers and are linked to the malignant transformation of tumors [123]. In a study related to glioma, GnT-III was found to play an anti-migratory role in α2,6-hyposialylated cells, and high expression of ST6GAL1 may weaken this effect and confer strong metastatic potential to cells, which may suggest that whether GnT-III is highly expressed together with ST6GAL1 may be a marker reflecting different degrees of tumor malignancy [107]. In addition, some isoforms in the GALNTs and protein (GalNAcTs) families have also been implicated as cancer-related biomarkers [124]. Mao et al. used Oncomine and TCGA database to analyze the transcriptional and survival effects of GALNT in pan-cancer and found that the expression of 13 GALNTs was associated with the prognosis of patients with low-grade glioma (LGG), suggesting that GALNT-related markers can be used as biomarkers for the identification of LGG molecular genotypes [125].

Significant changes in tumor cell surface glycosylation can provide an important source of markers for tumor progression, and abnormal glycosylated proteins can also be used as biomarkers. Geige et al. studied the expression of prostate stem cell antigen (PSCA), a highly N-glycosylated phosphatidylinositol (GPI)-anchored cell surface protein, in gliomas of different WHO grades and found that PSCA was absent in normal brain tissue and detectable in WHO grade III-IV gliomas [126]. Although weak PSCA protein expression was also found in some WHO grade I and WHO grade II tumors, PSCA expression was significantly lower than in WHO grade III-IV tumors [126]. Thus, PSCA may serve as a novel marker for WHO III-IV gliomas, while further studies are needed for its potential significance as a prognostic marker. Another highly glycosylated membrane-bound protein, the protein tyrosine phosphatase receptor zeta (PTPRZ), is mainly expressed in the CNS, where the glycosylated extracellular region is cut and shed, and the soluble cleaved form present in the cerebrospinal fluid (CSF) is called sPTPRZ [122]. Assessing the expression levels of sPTPRZ in CSF samples from patients with glioma, schwannoma, multiple sclerosis (MS), or nonneoplastic disease showed that sPTPRZ levels were significantly increased in CSF from glioma patients but not in CSF from other samples [122]. This result may illustrate the possibility of sPTPRZ becoming a diagnostic marker for gliomas, and sPTPRZ may even replace biopsy to differentiate glioma from other CNS diseases. Similarly, the integral membrane protein plasminogen activator urokinase receptor (PLAUR), which is highly glycosylated in gliomas, can be cleaved and released into blood and other body fluids and is closely related to prognosis. In other cancers, soluble PLAUR in blood can be used as a plasma marker of poor prognosis, but whether soluble PLAUR can be used as a marker of CSF or plasma in patients has not yet been validated [127]. As a multifunctional DNA and RNA-binding protein, nucleolin may be overexpressed in highly proliferating cells. It localizes predominantly in the cell nucleolus, and is also reported to localize in the form of phosphorylation/glycosylation on the cell surface [128]. Galzio et al. investigated the presence and localization of nucleoli in glioma specimens of different malignant grades and in gliomas cultured from surgically resected primary glioma cells, using an antibody against the gp273 protein that can recognize glycated surface nucleoli [128]. Their work showed that surface nucleolin increased with mounting malignant grades, suggesting its potential to be a histopathological marker of glioma grade [128]. As mentioned above, Bcan expression is upregulated in high-grade glioma cells, including GBM, whereas the Bcan isoform lacking most of the glycosylation, dg-Bcan, is only found in GBM tissues [129]. Based on this, dg-Bcan may have potential as a glioma-specific marker.

Alterations in glycosylation-related enzyme levels and significant changes in cell surface glycosylation in gliomas provide an important source of markers for their diagnosis, prognosis, and typing, and admittedly, a large variety of glycosylation-related biomarkers have been mentioned above; however, their specific reliability and feasibility on clinical examination are still being evaluated. In addition, although markers such as sPTPRZ and PLAUR have the potential to serve as CSF or plasma markers, a significant number of markers still need to be tested by invasive brain biopsy methods, and the ensuing potential complications, such as cerebral hemorrhage, and the length of time between sampling and diagnosis are also issues that need to be considered. In the future, the determination of the clinical utility of these potential markers needs to be repeatedly tested and carefully considered.

Tumor initiation, growth, and recurrence may depend on brain cancer stem cells (BTSCs), which can promote tumor invasiveness and may provide new therapeutic targets [130]. Glioma stem cells (GSCs) are a subset of proliferative tumor cells with self-renewal ability that can produce heterogeneous cells constituting tumors [131, 132]. Some hold the opinion that GSCs may be the basis of glioma development and resistance to existing therapies; [133] thus, it is also very important to study and compare markers of differentiated glioma cells, glioma stem cells and non-proliferating cells. CD133 is a cell surface N-glycosylated protein that can be salivated in neural stem cells and glioma initiating cells, [134] salivary acyl residues can be modified by α2,3-linked ligation to the N-glycosyl chain ends of CD133, and commonly used anti-CD133 antibodies, such as AC133 monoclonal antibodies (mAbs) that can be used to identify brain cancer stem cells in GBM and recognize glycosylated epitopes of CD133 [135, 136]. Because the glycosylation status of CD133 may vary at different stages of differentiation and in different tissues, CD133 is considered a marker for the isolation and characterization of normal and cancer stem cells [137]. However, this view is controversial, and another study found that different CD133 mAbs may recognize different CD133 splice variants with different glycosylated epitopes using different CD133 mAbs [134]. Different CD133 antibody clones will produce different findings, and differentially glycosylated CD133 can be detected on the membrane of differentiated tumor cells, while differential glycosylation may lead to specific epitopes being masked [138]. These factors make the reliability of the CD133 marker questionable. Considering the contradictions and controversies of the above results, C2E1 mAbs capable of binding full-length glycosylated CD133 on the cell surface were discovered in later studies and have been validated on GBM cells, updating the progress associated with GSC and CD133 markers [139]. Similar to the CD133 marker idea, because the known markers of cancer stem-like cells in solid tumors are cell surface glycoproteins and glycosylation, the posttranslational modification, is closely related to the expression of glycosylation-related genes, glycosylation-related genes may be markers associated with invasiveness or recurrence ability. Cheray et al. analyzed glycosylation-related gene expression during transformation between BTSCs and tumor-differentiated cells in GBM cell lines and selected eight genes (ATHL1, CHI3L1, GAA, KLRC3, GLT25D2, PRUNE2, ST3GAL5 and ST8SIA1) that could be used to characterize invasive and undifferentiated cells and speculated that some genes would have potential as prognostic markers [130].

The plasma exosomes secreted by cells have been suggested to contain a variety of bioactive N-glycoproteins that can be used as potential biomarkers for the diagnosis and treatment of early diseases, while the prerequisite for large-scale N-glycosylation profiling is the specific enrichment of N-glycoproteins/glycopeptides. Currently, a hydrazide-functionalized thermosensitive polymer has been developed that efficiently enriches and identifies protein N-glycosylation in human plasma exosomes by mass spectrometry. Quantitative comparison of this method revealed significant changes in 26 N-glycoproteins between glioma patients and healthy subjects, which also suggests the potential of this new strategy in N-glycoproteomic studies and biomarker discovery of plasma exosomes from glioma patients [140]. In addition, Raman imaging allows cellular interrogation and glycocalyx visualization without staining, providing important biochemical information. The results suggest that Raman imaging is robust for identifying structures and mapping attachment sites as well as glycan distribution in significantly heterogeneous tissues, while Raman imaging allows probing glycosaminoglycan distribution in healthy and cancerous tissues. The researchers showed Raman spectral features of GAGs in brain tissue and found that protein, lipid, and glycan metabolism was obviously dysregulated in malignant medulloblastoma [141]. This approach extends traditional biological approaches by identifying biomarkers based on unique vibration features.

### Therapeutic strategies

In previous studies, γ-secretase inhibitors (GSIs) have been shown to inhibit the Notch signaling pathway and attenuate CSC transmission potential in GBM,[142] while changes in glycosylation patterns have also occurred in GBM after drug treatment [143]. O-glycosylation has been suggested to be a critical part of Notch maturation during its arrival at the cell surface through the secretory pathway [144]. Thus, glycosylation may also be a research direction that can be considered for glioma treatment. The relationship between abnormal glycosylation in glioma and biological effects such as glioma cell proliferation, invasion, and adhesion has also been discussed earlier. Aberrant glycosylation is inextricably linked to the malignant behavior of gliomas, and conversely, many potential therapeutic strategies may be found from a glycosylation perspective. In the era of targeted cancer therapy, glycosylation has a significant impact on personalized cancer therapy, and glycan antigen-targeted therapy, specific targeting of protein glycoforms, glycan–lectin interactions, and interference of small molecules or specific glycan modifying enzymes with glycosylation pathways are important strategies for future glycosylation in cancer treatment strategy research [145]. At present, there are many perspectives on the treatment of gliomas, and they are still developing and advancing. This section focuses on glycosylation inhibitors, regulation of glycosylation-related enzyme expression, promotion of drug delivery and targeting, and other targeted strategies and related targets.

#### Glycosylation inhibitors

The link between the activation of RTK signaling and glycosylation in gliomas, particularly EGFR of the ErbB family of RTKs, has been discussed in previous subsections. This may be due to the fact that more than half of GBM-increased RTK signaling is mediated by amplification or mutation of the gene encoding EGFR [146]. Further studies have demonstrated that RTKs can also be used as a therapeutic target for gliomas. In glioma cell lines cultured in vitro, the use of nanomolar concentrations of the N-linked glycosylation (NLG) inhibitor tunicamycin decreased the expression levels of RTKs, such as EGFR, ErbB2, and ErbB3, significantly decreased RTK signaling through Akt, and radiosensitized tumor cells, perhaps due to inhibition of the synthesis of core glycans necessary to produce mature and functional RTKs [147]. Furthermore, glioma xenograft experiments in mice showed that inhibition of NLG in vivo could also reduce RTK protein levels in tumor cells and enhance the radiosensitivity of gliomas, and the constructed preclinical model integrating the ER-LucT NLG reporter gene that could noninvasively continuously image N-linked glycosylation in glioma cells also demonstrated the feasibility of targeted N-linked glycosylation in vivo [148]. Unfortunately, tunicamycin may not be a favorable and safe treatment for gliomas due to its cytotoxicity and narrow therapeutic window. In addition, although there are many related studies on RTK therapeutic targets in gliomas, and the efficacy of this strategy in the treatment of GBM is still limited by conditions such as tumor heterogeneity, signaling pathway redundancy, and the blood‒brain barrier [146]. In response to this current situation, it is proposed that therapeutic strategies with broad RTK inhibition may improve tumor heterogeneity and signaling pathway redundancy, leading to RTK targeting failure in GBM. In the past, clinical trials have found that GBM can undergo transcriptional deinhibition of PDGFRβ to evade EGFR tyrosine kinase inhibitors; that is, GBM can transform into PDGFRβ-dependent signaling growth through mTOR-dependent transcriptional deinhibition in the presence of EGFR inhibition, while combined abrogation of EGFRvIII and PDGFRβ can ultimately play a role in inhibiting GBM growth [149]. Based on this theory, Baro et al. investigated the effects of small molecule inhibitors of OST (NGI-1) on several RTKs (ErbB family receptors, MET, PDGFR, and FGFR1) using nanoparticle preparations validated by in vivo molecular imaging and clarified the effects of this treatment strategy that broadly inhibits GBM RTKs on increased effects of tumor cell radiosensitivity, chemotherapy-induced cytotoxicity, DNA damage, G1 cell cycle arrest and so on (Fig. 3b) [150].

#### Regulation of glycosylation-related enzyme expression

In addition to directly inhibiting abnormal glycosylation of proteins, changing the expression of related enzymes is also one of the ideas for the treatment of gliomas. As discussed above, FUT8 is involved in altering the fucosylation status of MET and EGFR, significantly activating the HGF/MET signaling pathway, and affecting the migration and invasion of glioma cells. Therefore, targeting FUT8 is also one of the pathways regulating RTK signaling, and studies have shown that this will have a synergistic effect with the first-choice chemotherapeutic agent in GBM, temozolomide (TMZ) treatment (Fig. 3b) [96]. In addition, Kroes et al. compared the gene expression profiles of normal human brain and gliomas and screened “sugar gene” targets with therapeutic potential. They found that the ganglioside-selective α2,6-sialyltransferase ST6 N-acetylgalactosaminide alpha-2,6-sialyltransferase 5 (ST6GalNAcV) expression levels were relatively lower in gliomas and glioma cell lines than in normal brain cells, while regulating the synthesis of glycosphingolipids on the surface of specific glioma cells may have a therapeutic effect on glioma invasiveness, which may also be one of the breakthroughs in the treatment of malignant brain tumors (Fig. 3b) [151]. Furthermore, they found that changes in the composition of specific membrane domains resulting from transfection of ST6GalNAcV in glioma cells affected their adhesion to fibronectin and laminin, increased HSP70 protein phosphorylation, and produced effects that reduced the invasive potential of glioma cells and inhibited tumor growth, which indicates that adjusting ST6GalNAcV expression in gliomas has therapeutic potential (Fig. 3b) [110]. Some drugs themselves also affect the levels of glycosylation-related enzymes, which can be considered as one of the related mechanisms of their efficacy. For example, As_2_O_3_ is effective in inducing apoptosis in solid tumors, and for gliomas, its possible mechanism is to reduce the expression level of β1,4GalT V β1,4-galactosyltransferase family, thus affecting β1,6-linked GlcNAc branch N-glycan galactosylation, reducing the expression of β1,6-GlcNAc-bearing N-glycans in cell surface proteins, and finally inducing apoptosis in glioma cells [152].

#### Facilitation of drug delivery and targeted therapy

Glycosylation is also the research direction of glioma treatment strategy because it can promote the drug delivery process, affect the interaction in targeted therapy and improve the stability of targeted drug delivery during the treatment. For example, human bone marrow mesenchymal stem cells (BM-hMSCs) have unique characteristics, including the ability to migrate or home and transplant into GBM, and have been investigated for therapeutic delivery in GBM, while their entry into tissues is largely dependent on glycosylation of glycan-glycans and glycan-protein adhesions between cells and the endothelium. It has been demonstrated that glycan composition differs between tissues causing homing of BM-hMSCs and tissues hindering BM-hMSCs in preclinical glioma stem cell xenograft (GSCX) models of GBM [153]. In another strategy, a mAb specific for the high-affinity interleukin-13 receptor α2 (IL13Rα2) targeted glioma cells expressing IL13Rα2 and improved survival of xenografted glioma tissues in nude mice, with the N-linked glycosylated portion of IL13Rα2 contributing to the interaction of the antibody and IL13Rα2 [154]. The mannose/glucose-specific lectin CaBo, which reduces cell viability and migration by inducing autophagy and cell death, can interact with glycosylated cellular targets and thus produce significant anti-glioma effects [155]. Similarly, dendrimers are multifunctional drug delivery platforms that can influence drug delivery strategies by targeting ligand modifications to influence receptor‒ligand interactions. Studies have shown that the coupling of β-glucose, β-D-galactose or α-D-mannose on PAMAM dendrimers confers their interaction with specific receptors, in which β-glucose modification significantly enhances the targeting of TAMs and microglia to regulate tumor immune responses by interacting with glucose receptors, increasing the penetration and cellular internalization of the blood‒brain barrier, while β-D-galactose-conjugated interactions with surface galectin affect the interaction between cancer cells and ECM [156]. In addition, in studies using glycopeptides as targeted parts of glioma drugs, the heptapeptide ATWLPPR (A7R) is considered to have a large potential for targeted delivery because it can specifically bind to vascular endothelial growth factor receptor 2 (VEGFR2) and neuropeptide-1 (NRP-1) overexpressed in glioma cells; however, glycosylated A7R derivatives have higher serum stability, cross the blood‒brain barrier more easily, and have stronger targeting ability [157].

#### Other targeted strategies and associated targets

Multiomics analyses, such as TCGA and the Repository for Molecular Brain Tumor Data Repository (REMBRANDT), have revealed many cancer-related potential molecular targets, and the application of these successful personalized medicine approaching in other cancers is often unsatisfactory in gliomas [146]. For example, bevacizumab, which inhibits RTKs, has shown promising anticancer activity in targeted therapy for colorectal cancer, but it has not improved overall survival in patients with newly diagnosed GBM in clinical trials [158]. Potential target molecules for glioma are still being investigated and updated to discover more therapeutic targeted strategies. Lee et al. projected a novel chimeric signal peptide-galectin-3 conjugate (sGal-3) that specifically targets abnormally N-glycosylated β1-integrins on glioma cell surface, which in turn triggers the oncoglycan-β1/calpain/caspase-9 pro-apoptotic signaling pathway to induce cell-specific death [159]. Studies have also been conducted on highly glycosylated PTPRZ in gliomas. The ligands pleiotrophin (PTN) and midkine (MK) inactivate PTPRZ, resulting in the inhibition of cell migration and tumorigenicity in turn, and their PTPRZ-selective, blood‒brain barrier-permeable mimetic molecules have also been developed and may contribute to the treatment of gliomas in the future [160, 161]. Furthermore, dg-Bcan may be used as a novel therapeutic targeted strategy for GBM in addition to glioma-related markers. Accordingly, von et al. designed a dg-Bcan-targeting peptide (BTP), BTP-7, for targeted therapy of GBM. Their findings showed that BTP-7 could be internalized by patient-derived GBM cells expressing dg-Bcan and had blood‒brain barrier permeability, and PET imaging demonstrated its targeting [129]. Similarly, the nucleolin gp273, which can be used as a biomarker, not only contributes to glioma grading but also may be used as a molecular target for therapy [128].

The application of glycosylation in glioma treatment strategies is discussed from the perspectives of glycosylation inhibitors, expression changes in glycosylation-related enzymes, promotion of drug delivery and targeted therapy, and potential molecular targets. In view of the link between abnormal glycosylation and the malignant behavior of glioma, the current research on glycosylation-related strategies is multifaceted and not limited to the four categories above. For example, Wen et al. tried to disrupt the interaction between neuron glial antigen 2 (NG2) chondroitin sulfate proteoglycan and galectin-3 to reduce glioma cell invasiveness, while N-linked glycosylation of NG2/D3 (D3 domain of NG2 core protein) was crucial in the interaction between the two [162]. Studies by Lopez Sambrooks et al. in EGFR-mutated non-small cell lung cancer (NSCLC) cell lines and xenograft mouse models have shown that the combined use of NGI-1, which partially disrupts N-linked glycosylation, will improve TKI (tyrosine kinase inhibitor) resistance and resensitize NSCLC to EGFR TKIs in EGFR TKI therapy [163]. The combination of glycosylation inhibitors and TKIs can also be used as one of the perspectives of exploration. In addition, some of the above treatment strategies only verify that they have a significant effect on glioma cells in vitro, and some studies have performed xenograft experiments. Meanwhile, their safety and efficacy in humans remain to be confirmed, and multiple animal experiments and clinical trials need to be supplemented from their formal application. Glycosylation-based treatment strategies will also receive more attention and be more deeply explored in studies targeting glioma therapeutic interventions.

## Conclusions

Overall, this article focuses on the evidence for aberrant glycosylation in glioma and its impact on tumor progression, related biomarkers and targeted therapeutic strategies. First, we summarized the research evidence that confirmed abnormal glycosylation in glioma cells and extracellular matrix based on the classification and specific process of glycosylation. Then, we investigated the specific mechanism of abnormal glycosylation affecting glioma progression from the perspectives of regulating protein function, affecting cell‒cell interactions, and triggering receptor downstream pathways. Its effects include promoting glioma cell proliferation, migration, and invasion, thereby enhancing the malignant characteristics of glioma. Finally, we discuss the utility of aberrant glycosylation in biomarkers and targeted therapies. In terms of markers, from the occurrence of altered protein glycosylation and abnormal expression of glycation-related proteins (e.g., glycosyltransferases) in gliomas, some proteins are expected to give some enlightenment for the advancement of novel diagnostic or prognostic biomarkers. In terms of treatment, this article discusses glycosylation inhibitors, regulation of glycosylation-related enzyme expression, promotion of drug delivery and targeting, and other targeted strategies and related targets. Although the current research on glycosylation and gliomas has involved many aspects and multiple angles, this topic needs to be more deeply explored compared with similar studies on other malignancies. For example, it is also necessary to enhance our thinking and understanding of (i) specific pathways downstream of receptors triggered by abnormal glycosylation; (ii) the link between glycosylation and glioma immunosuppression and evasion; (iii) how to apply the conjecture of glycosylation-related treatment strategies mentioned above to clinical practice; and (iv) how to break through tumor heterogeneity, signaling pathway redundancy and the blood‒brain barrier and strengthen the thinking and understanding of glycosylation-related glioma treatment and prognosis. With the development of data science, the existing glycosylation databases such as OGT-PIN and NetNGlyc can help the prediction of O-linked glycosylation and N-linked glycosylation modification sites respectively [164, 165]. Some researchers have applied NetNGlyc to the discovery of biomarkers for other malignant tumor, such as bladder cancer [166]. In addition, Qi et al. used TCGA and CGGA databases to identify the gene signature of gliomas, combined with their cohort and in vitro experiments, and then predicted the correlation between these gene markers and the prognosis and immune characteristics of gliomas [167]. All these above also indicate that the existing database is of great help to the research on glioma and glycosylation. Overall, increasing research has focused on the link between aberrant glycosylation and malignant features such as glioma cell proliferation, invasion, migration, and treatment resistance as well as clinical applications such as markers and targeted therapies. Glycosylation may play a major role in the innovation of effective treatment options for gliomas. Understanding the mechanistic basis of abnormal glycosylation affecting glioma progression not only helps to inspire researchers to further explore the relevant diagnostic and prognostic markers but also provides ideas for discovering effective treatment strategies and improving the survival and prognosis of glioma patients.

## Data Availability

Not applicable.

## List of Abbreviations

CNS: central nervous system
IDH: isocitrate dehydrogenase
GBM: glioblastoma
EGFR: epidermal growth factor receptor
GnT-V: N-acetylglucosaminyltransferase-V
GlcNAc: β1,6-N-acetylglucosamine
Asn: asparagine
Ser: serine
Thr: threonine
Cys: cysteine
LLO: lipid-linked oligosaccharide
ER: endoplasmic reticulum
GlcNAc-P: N-acetylglucosamine-phosphate
UDP-GlcNAc: uridine diphosphate-N-acetylglucosamine
Dol-P: dolichol phosphate
Man: mannose
GDP-Man: guanosine diphosphate mannose
OST: oligosaccharyltransferase
ECM: extracellular matrix
O-GalNAc: O-N-acetylgalactosamine
O-Glc: O-glucose
O-man: O-mannose
O-Fuc: O-fucose
EGF: epidermal growth factor
GalNAc-Ts: GalNAc-transferase
UDP-GalNAc: uridine diphosphate N-acetylgalactosamine
Gal: galactose
STn: sialyl-Tn
FUT: fucosyltransferase
POFUT: protein O-fucosyltransferase
GDP-Fuc: guanosine diphosphate-fucose
sLe(x): sialyl Lewis x
sLe(a): sialyl Lewis a
MALDI-MS: matrix-assisted laser desorption/ionization mass spectrometry
PNGaseF: peptide-N-glycosidase F
MALDI-MSI: matrix-assisted laser desorption/ionization mass spectrometry imaging
PGs: proteoglycans
GAGs: glycosaminoglycans
UAP1L1: uridine diphosphate-N-acetylglucosamine pyrophosphorylase-1-like-1
UAP1: uridine diphosphate-N-acetylglucosamine pyrophosphorylase-1
CSPGs: chondroitin sulfate proteoglycans
GPR56: G protein-coupled receptor 56
BEHAB: brain-enriched hyaluronan binding
DGC: dystrophin-glycoprotein complexes
DG: Dystroglycan
NEU3: neuraminidase 3
β3GnT8: β1,3-N-acetylglucosaminyltransferase 8
MMP-2: matrix metalloproteinase-2
ADAM: A disintegrin and metalloproteinase
PTPµ: protein tyrosine phosphatase type µ
RTK: receptor tyrosine kinase
GnT-III: N-acetylglucosaminyltransferase III
ST6GAL1: α2,6-galactoside sialylatransferase 1
RPTPs: receptor protein tyrosine phosphatase types
GALNT2: N-acetylgalactosaminyltransferase 2
LCA: lens culinaris agglutinin
MET: mesenchymal-epithelial transition factor
GOLPH3: Golgi phosphoprotein 3
PAI-1: plasminogen activator inhibitor-1
TCF: T-cell factor
FoxM1: Forkhead box M1
APC/C-Cdh1: anaphase-promoting complex/cyclosome
β4GalT5: β-1,4-galactosyltransferase 5
Hsc70: heat-shock cognate protein 70
TAMs: tumor-associated macrophages
LGG: low-grade glioma
PSCA: prostate stem cell antigen
GPI: N-glycosylated phosphatidylinositol
PTPRZ: protein tyrosine phosphatase receptor zeta
CSF: cerebrospinal fluid
MS: multiple sclerosis
PLAUR: plasminogen activator urokinase receptor
Bcan: brevican
BTSCs: brain cancer stem cells
GSCs: glioma stem cells
mAbs: monoclonal antibodies
GSIs: γ-secretase inhibitors
NLG: N-linked glycosylation inhibitor
NGI-1: N-linked glycosylation inhibitor-1
TMZ: temozolomide
ST6GalNAcV: ST6 N-acetylgalactosaminide alpha-2,6-sialyltransferase 5
BM-hMSCs: human mesenchymal stem cells
GSCX: glioma stem cell xenograft
IL13Rα2: interleukin-13 receptor α2
PAMAM: Poly (amidoamine)
A7R: heptapeptide ATWLPPR
VEGFR2: vascular endothelial growth factor receptor 2
NRP-1: neuropeptide-1
REMBRANDT: Repository for Molecular Brain Tumor Data Repository
sGal-3: signal peptide-galectin-3 conjugate
PTN: pleiotrophin
MK: midkine
BTP: Bcan-targeting peptide
NG2: neuron glial antigen 2
NSCLC: non-small cell lung cancer
TKI: tyrosine kinase inhibitor
